# Older People’s Experiences of Mobility and Mood in an Urban Environment: A Mixed Methods Approach Using Electroencephalography (EEG) and Interviews

**DOI:** 10.3390/ijerph14020151

**Published:** 2017-02-04

**Authors:** Sara Tilley, Chris Neale, Agnès Patuano, Steve Cinderby

**Affiliations:** 1 OPENspace, Edinburgh College of Art, University of Edinburgh, Edinburgh EH3 9DF, UK; A.Patuano@ed.ac.uk; 2 The Stockholm Environment Institute, Environment Department, University of York, York YO10 5DD, UK; chris.neale@york.ac.uk; steve.cinderby@york.ac.uk

**Keywords:** older adults, mobility, mood, built environment, mixed methods, qualitative, electroencephalography (EEG)

## Abstract

There are concerns about mental wellbeing in later life in older people as the global population becomes older and more urbanised. Mobility in the built environment has a role to play in improving quality of life and wellbeing, as it facilitates independence and social interaction. Recent studies using neuroimaging methods in environmental psychology research have shown that different types of urban environments may be associated with distinctive patterns of brain activity, suggesting that we interact differently with varying environments. This paper reports on research that explores older people’s responses to urban places and their mobility in and around the built environment. The project aim was to understand how older people experience different urban environments using a mixed methods approach including electroencephalography (EEG), self-reported measures, and interview results. We found that older participants experience changing levels of “excitement”, “engagement” and “frustration” (as interpreted by proprietary EEG software) whilst walking between a busy built urban environment and an urban green space environment. These changes were further reflected in the qualitative themes that emerged from transcribed interviews undertaken one week post-walk. There has been no research to date that has directly assessed neural responses to an urban environment combined with qualitative interview analysis. A synergy of methods offers a deeper understanding of the changing moods of older people across time whilst walking in city settings.

## 1. Introduction

As the proportion of older people in society grows, there is increasing concern regarding poor mental health in old age [1]. A 2004 survey on the mental health and wellbeing of older people in Scotland found that 10 per cent of people aged between 60 and 74 years had a common mental disorders such as anxiety or depression [2]. It has been estimated that poor mental health in older age will cost the UK’s National Health Service in excess of £34.7 billion annually by 2026 [3].

Continued mobility in older age plays a vital role in improving quality of life by facilitating independence and social interaction and thus reduces isolation and loneliness, which is a key concern in older age [4]. Psychological benefits from being “out and about” [5], particularly through “discretionary” or “on a whim” trips [6], potentially have an important role to play in the prevention of poor mental health. Therefore, everyday mobility choices may have a crucial role to play in the wellbeing of older people, as well as in lifelong health and wellbeing trajectories.

Walking as a mode of transport and form of physical activity is something in which the majority of people are able to participate. Walking amongst older people is increasingly considered favourable as a mode of transport as it is, arguably, safe and accessible and can be built into everyday routines [7]. However, the built environment needs to be supportive, such as having seating provision readily available, if it is to enable older people to be mobile outdoors [8]. There may be opportunities for aspects of the built environment to improve mood. There are a large number of studies that suggest that natural environments are preferred over urban environments [9,10]. Walking in green spaces has been shown to be beneficial for both wellbeing [11,12] and cognition [13,14], as well as benefiting older people in urban spaces [15,16]. Attention Restoration Theory (ART; Kaplan [17]) posits that an individual’s surrounding environment impacts on their attention. Environments that demand focused or directed attention, such as busy urban spaces, lead readily to mental fatigue [18]. Natural environments, however, are more effective in offering psychological restoration and relief from fatigue [19,20]. Research has shown the beneficial effects of green spaces on cognitive performance aligned with the ART model [13]. The positive effects of nature have also been shown in clinical populations, such as those with depression [14]. Recent laboratory-based neuroimaging studies have shown that different environments may be associated with distinctive patterns of brain activity [21,22,23,24,25]. Further research has shown differences in neural activity derived and interpreted via Emotiv electroencephalography (EEG) proprietary software when walking in various urban environments, indicating changing neural activity in response to changing urban environments [26].

There has been no research to date that has directly assessed interpretations of EEG responses whilst walking in an urban environment combined with qualitative interview analysis to assess changing mood. A unique aspect of the collection of EEG data is that it allows for uninterrupted objective data collection from participants on a second-by-second timescale. However, interviews are important to gather subjective responses to the environment and assist in interpreting EEG responses such as those offered by Emotiv software. Interviews using video elicitation can also prompt discussion and stimulate recall of particular events, such as those that occur during walks, and therefore offer insights to participants’ experiences [27]. The combination of assessing EEG-based data with qualitative data is underutilised at present. This study pilots a novel methodology in order to begin to address this gap in the literature. The EEG system described in this study has been used to gather data on environmental effects during previous walks [26], but a definitive set of self-reported experiences to underpin interpretations of the EEG data is currently lacking.

This study is part of a wider research project exploring older people’s responses to place and their mobility in the built environment. The aim of this paper is to understand how older people experience different urban environments and how this impacts on their mood, using interpretations of EEG along with subjective scales and retrospective interviews. It is unclear if there are age related changes related to the restorative effects of green space. Neuroimaging, studies, including EEG, undertaken in laboratory settings lack ecological validity as these settings do not incorporate smells or sounds, for example. This study seeks to build upon existing limited research that uses mobile EEG in real world settings, using qualitative methods to capture participants’ subjective feelings to assist in interpreting the EEG data. Therefore, this study uniquely combines quantitative software interpretations of EEG outputs, subjective reports, and qualitative interviews. The EEG output provides a real time psychophysiological measure of response to a changing urban environment, while the self-report measures offer a context and understanding to these changes. This paper will also reflect on the methodological considerations for further research.

## 2. Materials and Methods

### 2.1. Participants

**Figure 1:**
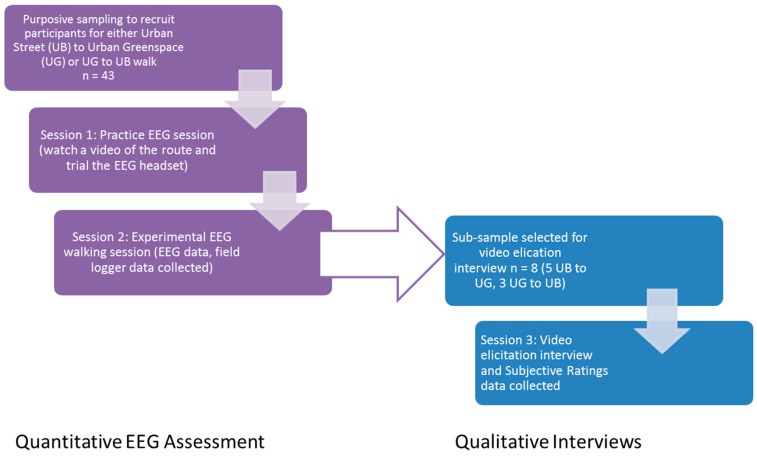
Flow diagram of study methods and sample size.

Purposive sampling was used to recruit 43 participants aged 65 years and over to walk a continuous route that was comprised of an area of green space and a busy urban street in Edinburgh, UK, while wearing an Emotiv EPOC + EEG headset. A week later, interviews were completed with eight participants, which was 19% of the sample (*n* = 8, mean age = 75.75 years, standard deviation = 6.76, range = 67–86 years). These eight participants made up the study population that is discussed in this paper. Due to participant fatigue and time constraints only a small sample of participants could take part in a follow up interview. Initially 10 participants were recruited, but two people subsequently declined to be interviewed due to time constraints. A total of three men and five women took part in the interviews. Figure 1 presents a flow chart of participant recruitment, sample size and the sessions they were involved in.

All participants were required to be able to walk unassisted by another person for at least 15 min. Participants were also screened over the phone prior to Session 1 to ensure that they understood the study and also did not fall into any exclusion criteria. During the screening, participants were asked if they had any visual impairments, chronic mental illnesses, or a history of epileptic or psychiatric disorders, as well as confirming that they could walk unassisted for 15 min, all of which make up the exclusion criteria of the study. If they did not meet these criteria, then they would not be considered for the subsequent testing sessions. Ethical approval for the study was provided by the Edinburgh College of Art Ethics Committee, University of Edinburgh (ref. 09/04/2015). To account for brain hemispheric differences [28] all participants in this study were right handed.

### 2.2. Experimental Design and Procedures

This study was conducted between May and September 2015 (excluding August 2015 when the Edinburgh Festival takes place). Participants took part in three sessions over the study period, as outlined in Figure 1. The first two sessions (a practice session and an experimental session, roughly a week apart) involved wearing the EEG headset and a data acquisition computer in a backpack and were carried out over two separate mornings (between 9:30 am and 12 pm) to ensure that participants did not suffer from time of day effects [29,30]. While we could not control the weather conditions, we ensured that participants only went out on dry days free from high winds or rain (but conditions could be cloudy, as long as they were dry). The practice session, undertaken after screening, served as an opportunity to demonstrate the EEG headset and familiarise participants with the route they would take (both described below) during the experimental session. This was achieved by showing participants a video of their given route lasting roughly 15 min.

During the experimental session, participants were instructed to walk their assigned route at their own pace, followed by a research assistant walking behind who made field notes and recorded any unusual activity or events and who was also present for health and safety purposes. The research assistant did not speak to or walk alongside the participant during the study, except if asked for instructions by the participant. Following the completion of the walk, participants were asked about their experience of the walk during the experimental session, including their impressions of the environment as a place to walk, whether they were familiar with the area, and how they felt during the walk. Data was also captured on how often they walked alone.

During the walk, a GPS recorder was used to track the timing of the walk and a smartphone app, Fieldworker [31], was used to record any events during the walk, e.g., if the participant asked for instructions during the walk, which were then time-stamped and linked to a GPS location. For this analysis, the events were grouped into 8 time segments and the number of participants it affected noted alongside.

Table 1 summarises the weather conditions during the walk outside and also considers whether participants had ever walked all or part of the specified study route before.

Five of the participants had walked at least some part of the route prior to completing the study. The other three participants had never walked the route before. Participants were also asked if they were familiar with the surrounding study site area more generally. Half of the participants were “slightly familiar” with the study site area, with one participant being “moderately familiar”, whilst the remaining three participants were “not familiar” with the study site. Almost all of the participants found the walk “easy” or “very easy”; to complete, with only one participant responding that they had found the urban busy section of the walk “difficult”. All of the participants walked alone, with six stating that they either “always” or “very frequently” walked alone and two participants stating that they “occasionally” walked alone.

All but one of the participants walking from the urban busy section into the urban green experienced sunny and warm weather. For those completing the urban green to urban busy walk, two participants experienced cloudy and cold weather, whilst one had sunny and warm weather for their walk.

### 2.3. Routes

The study site was in Leith, Edinburgh, UK, which is the most densely populated district of Edinburgh. The 2011 Census recorded almost 26,000 inhabitants spread within an 800 m radius of Leith Walk, the main axis of which links Leith to Edinburgh City centre [32]. This site was selected due to the proximity of green to urban busy space as well as a reasonably flat gradient to ensure that older participants could undertake the route without excessive exertion.

**Figure 2:**
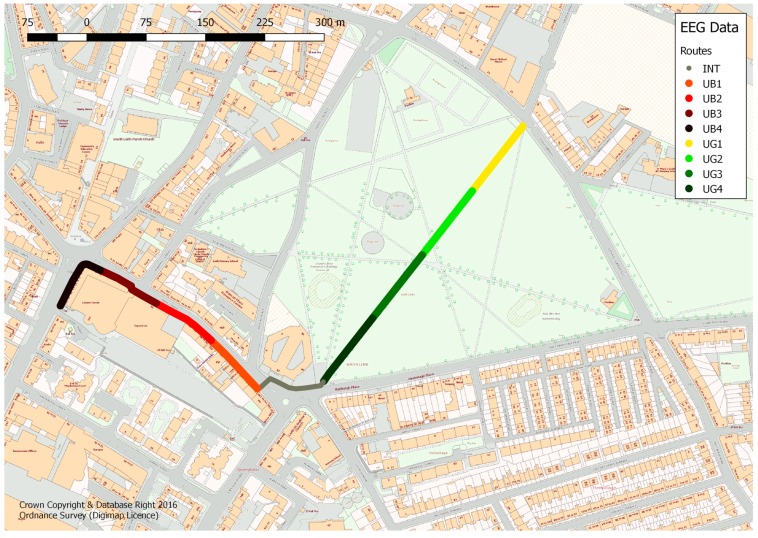
Map of the walking route undertaken by participants (walking in one of the two possible directions). Note: UG = Urban green (green space); UB = Urban busy (streetscape); Int = Interchange, the transitional section between these two environments.

**Figure 3:**
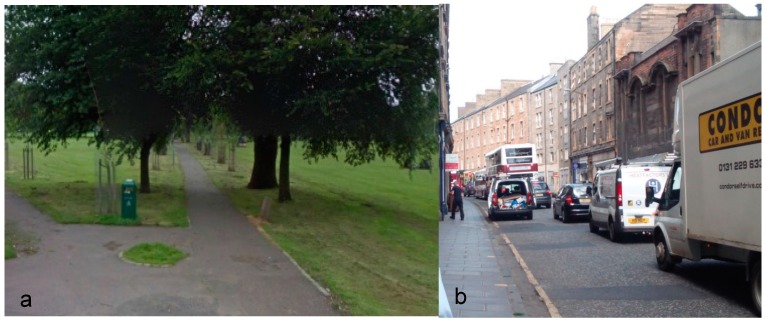
Street views of the two walking environments; (**a**) urban green and (**b**) urban busy (photo credit: OPENspace Research Centre).

Participants walked along one of two routes, as indicated in Figure 2, taking in two urban environments; urban green (Figure 3a) and built urban busy (Figure 3b). Participants were required to walk either from the urban busy into the urban green space or vice versa, with the design being counterbalanced to understand if there are any neural or subjective responses that are typical of a given environment irrespective of what order they are presented in. Figure 2 also indicates an interchange area (in grey) between the green and urban busy space, which was removed from the analysis to ensure responses referred to just urban green or urban busy conditions. The green space section is a public park, which is a largely flat expanse of grass bordered by mature trees and covering 19 ha. The park also encompasses a central path 0.3 miles long, which is lined with trees. The urban busy section is around 0.2 miles long, with quite narrow street paving and bordered by relatively high buildings (mostly 5 storey apartment buildings) and shops, including a large supermarket. Between 2005 and 2014, there were 22 road accidents on this stretch of road [33]. Of the pedestrian casualties that have occurred, eight involved pedestrians, all of whom were below 65 years old. Table 2 provides a typology of the different environments.

### 2.4. EEG Data Acquisition and Analysis

Brain electrical activity was recorded non-invasively from the scalp using the Emotiv EPOC+ EEG headset with 14 channels corresponding to the international 10–20 position system (AF3, AF4, F3, F4, F7, F8, FC5, FC6, T7, T8, P7, P8, O1, and O2). P3 and P4 acted as reference electrodes. Electrode impedances were kept below 5 kΩ and signals were internally sampled at 1024 Hz before being filtered and down sampled to 128 Hz per channel and sent via Bluetooth to the acquisition computer. For this analysis, we used output from the Affectiv Suite, proprietary software developed by Emotiv, to deduce emotional reactions from brain activity. The Affectiv Suite creates a different profile for each individual to account for potential differences at the neural level and then interprets the EEG activity from the available channels into four emotional parameters; “excitement” (short term arousal), “frustration”, “engagement”, and “meditation”. These parameters were normalised for each individual and scaled as values between 0 and 1, which allowed between subject comparisons, at each sampling point. This process results in approximately seven samples per second (7 Hz).

Due to the intellectual property rights of Emotiv, it is unclear what particular EEG signature underlies each of the Affectiv Suite outputs. Based on findings from previous research [26], “engagement” appears to be associated with arousal and “excitement” with directed attention, and “frustration” has negative valence, while “meditation” is associated with a calm relaxed state. Due to technical issues resulting in poor data quality, the “meditation” channel was not analysed in this study; therefore only the “engagement”, “excitement”, and “frustration” parameters were used. The EEG data set was generated by creating four sequential means per walking segment at the individual level and then averaging these means across the whole cohort for each walking route. These means were generated using the time taken for a given participant to complete a particular section of the walk and then dividing this into four time locked sections. It is these means that are used for the analysis of the Affectiv Suite data presented in this paper.

### 2.5. Video Elicitation Interviews

A week later, the eight participants selected for a follow-up interview were asked to watch a pre-recorded 15-min video of the route that they had previously walked. The video was paused at 4 equal intervals per environment type (i.e., urban busy and urban green and including the interchange period), creating 9 video segments. During playback of each segment, participants were asked to describe their remembered experience of the walk, including what happened during the walk, what they noticed during the walk and how they felt. Field notes from the walk and the GPS event logger were available to the researchers as prompts for the walk and could assist the participants in remembering the walk. At the end of each video segment, participants completed a 10 cm visual analogue scale (VAS) indicating how “excited”, “engaged”, and “frustrated” they felt whilst walking, corresponding to time matched Affectiv Suite outputs. The VAS was measured and scaled from 0 to 1 to correspond with the Affectiv suite outputs. This assessment was conducted a week later to minimize participant fatigue on the testing day and also because we did not want to interrupt the participants during the EEG recording as this would affect the quality of the data. The interview also provided the participants with the opportunity to discuss these terms.

Interviews were recorded and transcribed, and inductive thematic analysis, a widely used qualitative analysis method [34] was carried out on the transcripts. This involves establishing themes through patterned responses in the transcripts as opposed to establishing a coding framework prior to conducting the analysis. To undertake this process, transcripts were analysed by the direction of the walk undertaken to provide context for the EEG analysis as well as to consider if participants had different experiences depending on the environment in which they started the walk. To consider older people’s interpretation of the Affectiv Suite terms, the sections following each segment where participants were asked to complete a VAS scale were also analysed separately. The full transcripts were also examined to identify themes that emerged for all participants, regardless of the direction walked. Analysis began with initial coding, which was then organized into different themes. For the Affectiv Suite terms, the themes included positive and negative interpretations, appropriateness, and questioning the terms. The themes for all participants, irrespective of the direction of the walk, included feeling anxious at the start of the walk, awareness of study conditions, navigation and wayfinding, familiarity, social interaction, and weather.

## 3. Results

This section first presents the findings from the participants that started their walk in the urban busy environment and then walked into the urban green section. The second section presents the findings from the participants starting the walk in the urban green environment and then walking into the urban busy section.

### 3.1. Urban Busy to Urban Green Analysis

**Figure 4:**
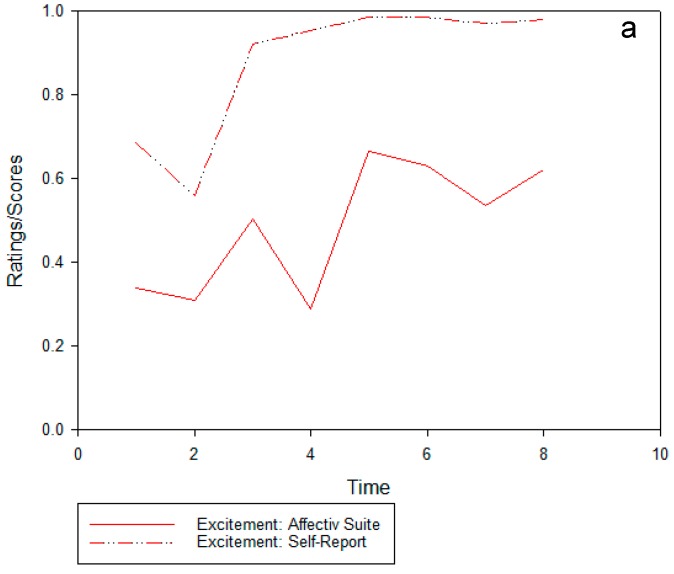

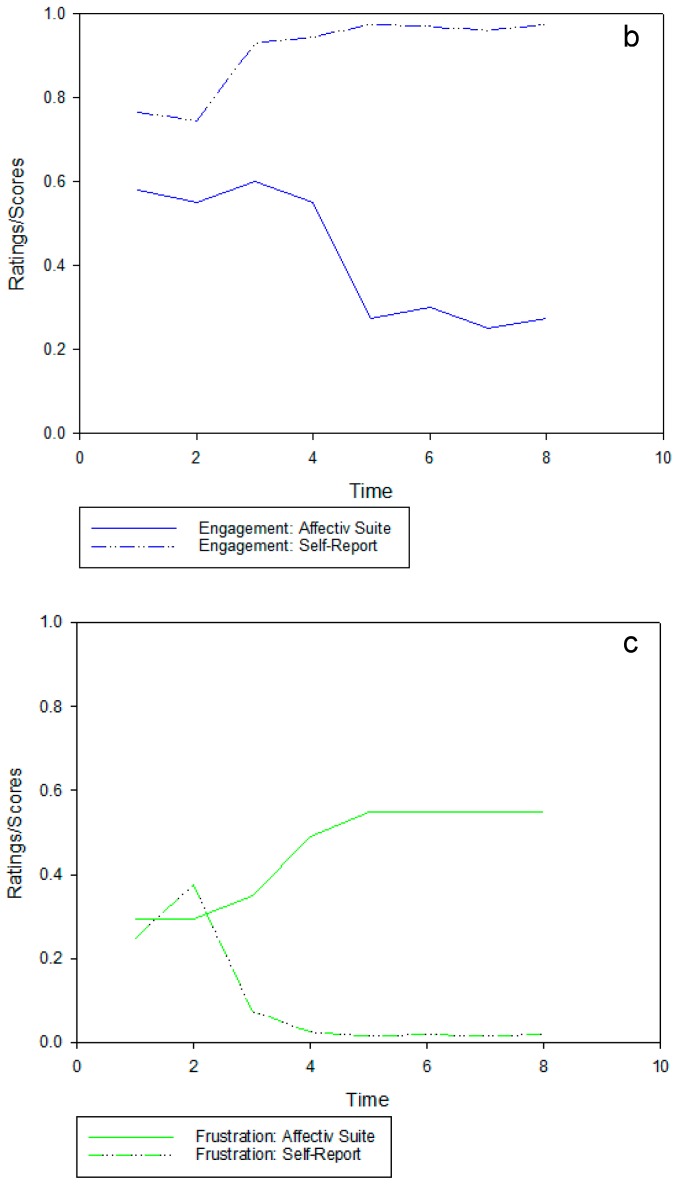
Affectiv suite and self-report outputs ((**a**) “excitement”, (**b**) “engagement”, and (**c**) “frustration”) during the urban busy to urban green walking route. Time points 1–4 refer to the first section of the walk (urban busy) and time points 5–8 refer to the second section (urban green).

Figure 4a–c shows the three Affectiv Suite channels, “excitement” (Figure 4a), “engagement” (Figure 4b), and “frustration” (Figure 4c), with corresponding self-report ratings of the same terms.

Overall, there is a similar pattern between the Affectiv Suite and self-report ratings for the first three time points in the urban busy walk for the “excitement” channel in Figure 4a, but there is a magnitude difference whereby participants rate their excitement levels higher than the Affectiv Suite outputs across all time points. The “engagement” and “frustration” channels do not appear to align with the Affectiv Suite and self-report outputs beyond the first three time points in the “engagement” channel and two time points in the “frustration” channel.

Table 3 presents the number of different events that occurred during the walk for the group of participants undertaking the urban busy to urban green walk. More “events” occurred in the Urban Busy section with obstacles such as pedestrians, street furniture, or construction works.

Table 3 provides additional context for explaining the Affectiv Suite results. Focusing on the “engagement” channel, participants starting their walk in the urban busy were concerned with a number of obstacles. As participants moved into the urban green space, the “engagement” channel declined, which might indicate that people were more alert in the urban busy section as they navigated the path and associated obstacles, but they then relaxed as they entered the green space and the number of obstacles was reduced.

The interview analysis helps to explain the divergence of the self-reported data from the Affectiv Suite data. During the interview, participants commented on the terms used on the self-report scale relating to those used in the Affectiv suite software. In particular, there was some questioning of the use of the “engagement” term amongst participants:

“I’m not quite sure what you mean by engaged.”

“Engagement” was also interpreted as being both positive and negative. Participants mentioned that they were engaged with the experiment but were also engaged with the environment, largely in terms of navigating the pavement in order to avoid other pedestrians, as well concentrating on uneven paving. However, they were also engaged with the green environment, particularly the green scenery:

“I don’t know how I could say I was engaged. I was engaged in watching where my feet were going, because the pavement was very rough in parts.”

The differing interpretation of “engagement” in the urban busy to urban green walk can be seen in Figure 4b where participants’ self-report ratings of “engagement” follow a similar pattern to the Affectiv suite in the urban busy stage, albeit at a different magnitude, before becoming markedly different towards the end of the urban busy walk in the urban green walk.

“Frustration” was also a term that participants appeared to interpret differently, as it did not relate to their immediate experience of the environment during the walk:

“If you remember, my engagement and frustration are all about social economic planning, you know.”

The “frustration” channel shows large differences between the Affectiv output and the self-report ratings after an initial period during which ratings and outputs appeared to align. Levels of “frustration” appeared to increase going into the urban green walk in the Affectiv channel, while self-report measures suggest “frustration” levels dropped going into the urban green walk.

In the “excitement” channel, there appears to be a similar pattern between the self-report and the Affectiv responses, but, as with the “engagement” channel, the magnitude of these changes is noticeably different. During the interview, the participants did not question this term as much as the other Affectiv Suite parameters.

The video elicitation interview analysis provides further context to the EEG data. Participants that started their walk in the urban busy environment had negative views of the area:

“It was very unpleasant, noisy, dirty, lots of litter lying around.”

Some participants were watching the pavement a lot for uneven paving and were anxious about falling during the walk. There were also vigilant in watching other people on the narrower pavement, which also made them feel anxious:

“Then you went on to a more, sort of, narrow pavement and then there’s these, whatever they are, the signs just at the moment to say there’s all of this happening. So you were dodging people and watching as you were going along.”

During the urban busy section, participants had a heightened awareness of the traffic and other pedestrians they saw, especially navigating the path, which would be in line with the Affectiv Suite EEG data and self-reports.

As the participants approached the interchange between environments, they mentioned they felt their mood improve when they saw the researcher in the middle of the route to direct them to the green space section of the walk. Upon entering the green space, participants felt more relaxed and peaceful. Also they felt relieved at having left the urban environment, which is also in line with the interpretation of the Affectiv Suite EEG data and self-reports:

“By that time I was actually getting quite happier (sic) because I could see (the researcher) across at the lights. I thought, right, I’ve just got that wee bit to go then I stop at the lights, thank God.”

The weather had an impact on the participants’ mood, with one participant noting that the grey weather had a negative impact, whilst another felt positive at the start of the walk when it was good weather:

“Well, it was a, sort of, misty day, a bit dreich (overcast) and it was…yes, people were just, sort of, coming towards you and I didn’t feel really anything. I didn’t feel too embarrassed but yes, I think it’s a grey area so therefore it makes you feel a wee bit grey.”

### 3.2. Urban Green to Urban Busy Analysis

**Figure 5:**
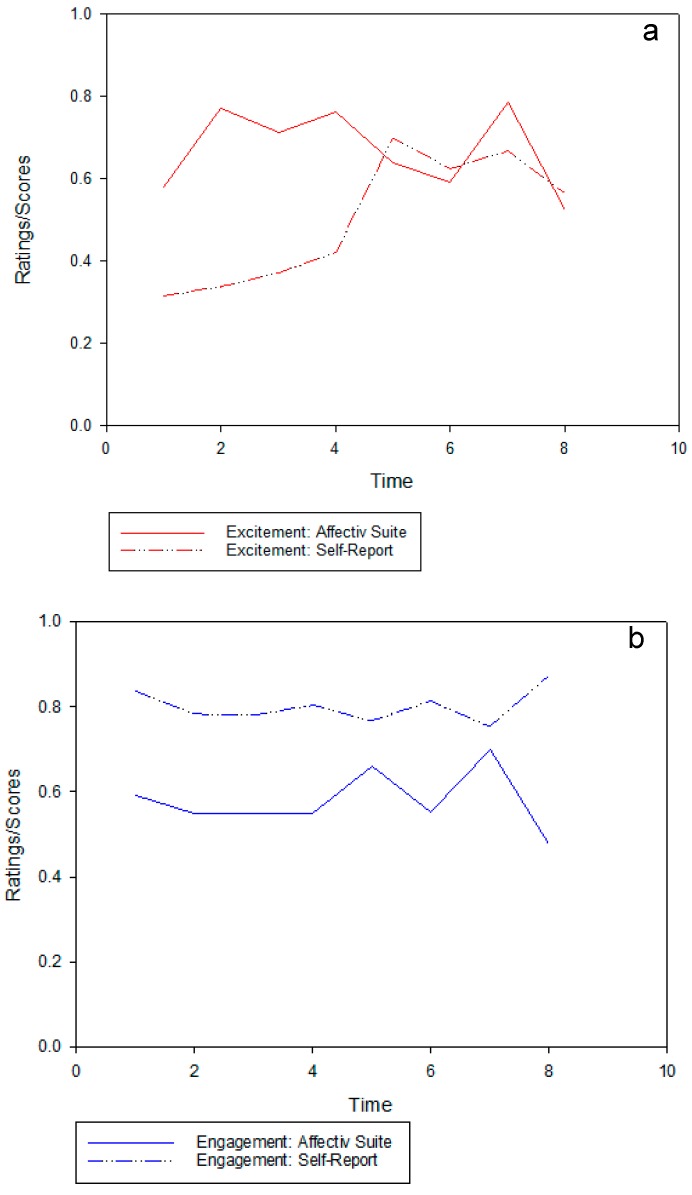

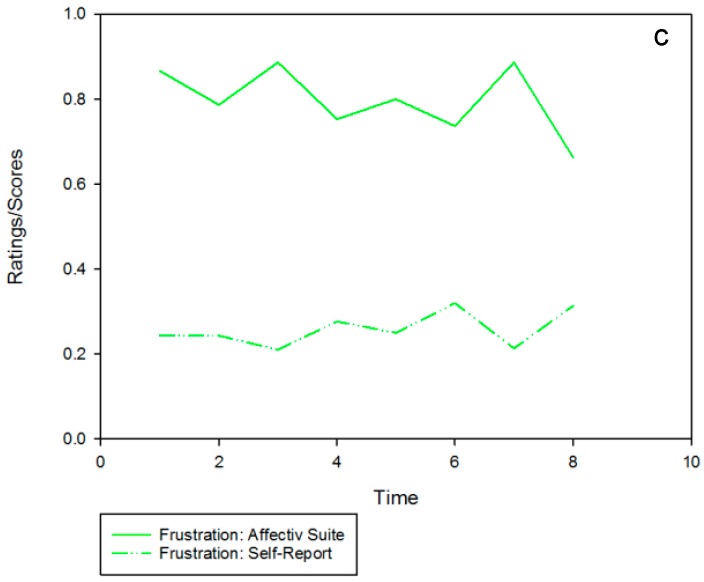
Affectiv and self-report outputs ((**a**) “excitement”, (**b**) “engagement”, and (**c**) “frustration”) during the urban green to urban busy walking route. Time points 1–4 refer to the first section of the walk (urban green) and time points 5–8 refer to the second section (urban busy).

Figure 5a–c shows the three Affectiv channels, “excitement” (Figure 5a), “engagement”, (Figure 5b) and “frustration” (Figure 5c), with corresponding self-report ratings of the same terms.

In Figure 5a, while there does not appear to be much alignment between “excitement” levels in the green section of the walking route, there is some alignment between the Affectiv output and the self-report ratings in the built urban section of the walk. The “engagement” channel in Figure 5b shows a similar pattern, despite some incremental changes towards the end of the walk, between the Affectiv output and the self-reported ratings, but the magnitudes of these ratings are very different. The “frustration” channel in Figure 4c shows similar patterns between the Affectiv and the self-report ratings. However, the levels of “frustration” are reported by the participants to be much lower than is shown in the Affectiv output.

Table 4 shows the number of events that happened during the walk for the group of participants undertaking the urban green to urban busy route. Also shown in this table is that the number and frequency of “events” occurring in the urban busy section is higher than in the urban green section. However, for this group of participants, these events were the points at which the participant(s) asked for instructions to follow the route. However, there was also a presence of loud noise in the green space defined by the researchers as extreme, indicating that it wasn’t a normal urban green experience. This could refer to an emergency services siren or construction works, but the exact source of the noise was not recorded.

The “excitement” channel in Figure 5a shows a large difference within the urban busy space, with the Affectiv output suggesting that participants showed a higher level of “excitement” in the urban green space than what they reported in the video elicitation interview. In the urban busy walk, the Affectiv and self-report outputs appear to align within the same scores. However, as “excitement” for self-reports increased, it may be indicative of participants being more alert, although they questioned this term and found it difficult to interpret for the study:

“So when you’re saying excited, do you mean that I’m thinking less about being in a nice, pleasant…nice about this being a very, very pleasant experience I’m thinking, I’m going to have to be careful; I’m going to have to be alert and aware?”

Participants report being more engaged throughout the walks than is shown in the Affectiv output in Figure 5b. Participants walking from the urban green space to the urban busy environment started the walk with engagement at the same magnitude as participants walking the other route. However, they remained at the same level of engagement throughout the walk, suggesting that they had to stay alert in the urban busy space to navigate the route. Table 4 indicates that participants required more assistance in navigating the urban busy section. The participants questioned the use of the “engagement” term, although more negatively compared to those walking the other route. “Engagement” tended to be interpreted by participants as referring to alertness, in particular to the environment, as they were watching out for other pedestrians as well as being attentive to the study protocol. As one participant mentioned during the urban busy section:

“I wasn’t threatened by the people but it’s just quite a narrow pavement, so I’m certainly engaged.”

In Figure 5c, the “frustration” channel shows a similar pattern between the Affectiv and the self-report rating. However, levels of “frustration” are reported to be much lower than is shown in the Affectiv output. During the interviews, participants found the green space relaxing, discussed further in the following section. However, the self-reported “frustration” slightly increased in the urban busy section, although the participants questioned the use of this term. As one participant reasoned when rating frustration in the urban busy section:

“So frustration, to the extent it’s less pleasant.”

Turning to consider the themes arising from the video elicitation interviews, further contextual data, beyond reporting on the Affectiv-labelled responses, helps in interpreting the results. Participants starting the walk in the urban green space found this environment relaxing, peaceful, and enjoyable, with people taking interest in the nature of the park and other people such as dog walkers using the space. Being away from the traffic also helped the participants feel relaxed:

“It was quite nice at that point and quite peaceful really because it’s away from the traffic and noises.”

As they started walking into the urban busy environment, the participants reported feeling more aware of other pedestrians and traffic. They also reported increased anxiousness due to the visibility of the headset upon approaching the urban area:

“I felt a bit more self-conscious, by this time, I think, you know, a bit more people around, and worried what they were gonna...what I looked like with this headset on.”

In the urban area, participants noticed buildings and other points of interest that were familiar from the video shown during the practice session, which they used for wayfinding. They were also more aware of navigating the path as it became narrower and busier with other pedestrians. They also felt that the area was more deprived in terms of how the environment looked:

“I enjoyed walking in the green space much more than I enjoyed negotiating the hazards of the traffic and working out how I’m going to pass the people who were coming towards me, and I remember thinking, people look more poor here than they do in my area.”

### 3.3. Additional Video Elicitation Interview Themes

During the interviews, all the participants were confidently able to remember their experience during the study. There were some common themes across all participants, regardless of which walk that they did. These findings may assist future EEG studies with older participants and/or where mixed methods are used.

Many of the participants we interviewed reported feeling nervous and anxious at the start of the walk, due to the visibility of the headset, regardless of which environment setting they began the walk in. However, most people eventually forgot about the headset and were surprised they didn’t receive more looks or attention from pedestrians. However, participants noted that, whilst they were largely ignored by pedestrians, they did notice people observing them from the bus:

“I felt very apprehensive and a bit silly…Well, it was just the thought of people watching me but the problem wasn’t actually people watching me as pedestrians, it was actually people on the bus and they were sitting looking down on you when they were stopped. So that was the part I actually felt more uncomfortable at.”

Although participants expressed this anxiety, they still carried on taking part in the project as it was seen as a “good cause” that they were helping in contributing to knowledge. Some also saw it as “a job to be done”, so they were aware and interested in the study. However, nearly all of the participants forgot that they had the headset on towards the end of the walk:

“When (the researcher) put it on in the car park I said to her, oh, good grief I didn’t realise I was going to have this on. Once I got it on and went into the street I thought, what the hell. I thought, if you’re helping somebody or something, and I forgot all about it. I really did. It wasn’t uncomfortable. In fact, I forgot it was on to be quite honest.”

As a result of being aware of the study, it appeared that some of the participants were considering the outcomes of the study as well as being concerned about the equipment, especially the computer that they carried during the walk and whether it was working:

“Well you know because you do think you’re part of an experiment and I’m sure that that changes the nature of any walk, but how can you gauge how I feel without taking part in the experiment, but I enjoy walking in green places and I remember looking at all these flowers and thinking, it’s beautiful.”

One participant said that the computer/tablet became heavier towards the end of the walk. Care was taken to ensure that the computer was as light as possible for older people to carry. However, for some older participants such equipment may still be heavy to carry. As technology develops, there may be different options to undertake such research.

Participants were also very aware that they needed to follow a particular route during the study, made familiar to them by watching a video of the route during the practice session. It appeared that participants had noted particular landmarks and other points whilst watching the video prior to the walk to help them navigate the route. As a result, during the walk on site, participants were looking around for familiar point:

“My eyes were looking for the yellow door, from the film I’d seen the week before, there was a yellow door somewhere...there you are. That was something I had in my head, I thought, look for a yellow door.”

Participants were also told that a researcher would be at the middle of the walk to ensure they continued along the route correctly. This also provided a “landmark” for the participants to focus on, and all the participants reported during the interview that they were actively seeking out the researcher in the middle of the walk:

“Well, I was looking, I was looking for (the researcher). Because I’d been told I would meet her. And I saw her at the other side of the crossing.”

During the walk, participants that were familiar with the area would focus on and/or think about things that were familiar to them; for example, historic buildings and particular buses that they recognised. These were things that they had seen previously or had heard about in the past or from other social contacts. The participants reported feeling happy when they were reminded of good memories they had of this particular environment.

The weather on the day and social interaction along the route also mediated the participants’ experience of the walk. In relation to the weather, warm and sunny weather led to participants reporting being in more positive mood, which was further improved by seeing more people outside. However, those walking on days when the weather wasn’t sunny did not report similar feelings:

“The weather, undoubtedly, was a help. If that had been a bad day, I probably would have been ticking words like grumpy (on a mood scale), or whatever! But how could you be grumpy on a day like that, it was gorgeous.”

Interaction with others seemed to be important in the built environment as it brought joy to the participants:

“And I met, only two people, two women, and they both greeted me, which was very pleasant.”

However, a lack of interaction led to them feeling down, particularly if participants reported that they usually were the ones to greet other people. As one participant put it:

“Yes, I just felt it was miserable and I didn’t feel it was friendly, how some places you go and somebody says, good morning, hello, and that but there didn’t seem to be any.”

During the walks, participants also mentioned that they frequently watched paths and paving for uneven surfaces, particularly if they had a walking stick. The route selection had taken into consideration the gradient of the route to ensure participants could undertake the route without excessive exertion and to ensure EEG changes were due to environmental change and not change in walking gradient [35]:

“I was just watching where I was going and watching for paving slabs sticking up.”

The interviews also provided an opportunity to ask about particular behaviours participants displayed during the walk (e.g., stopping to look at things), as they could not be disturbed during the walk and researchers may not have wanted to make participants feel uncomfortable at the end of the walk by asking them about it straightaway.

(Interviewer) “The researchers noted that you stopped and had a little look at (a plaque in the park). Is that any relevance to you at all or is that just something that you thought might have been interesting at the time?”

(Participant) “What I did think, now I’m remembering, what an insignificant looking little thing. To me it should have been more prominent. Yes, it was like somebody had just laid it there and moved on.”

## 4. Discussion

Due to the change in Affectiv outputs and self-reported scales observed during the study, the results show that participants experienced varying mood, measured as levels of “excitement”, “engagement”, and “frustration”, in different urban environments whilst walking. This was further reflected in the themes that emerged from the transcribed interviews. However, the levels of different mood states varied according to the different methods used to measure them, suggesting that a synergy of methods may offer a deeper understanding of the changing moods of older people across time whilst walking in city settings. The self-report measures and interviews provide context for the neuroimaging data. Furthermore, as has been shown previously [10], there appears to be a preference for the green space, this time in an urban context, amongst the older participant group involved in the study.

There were some aspects of the built environment along the route that led to both positive and negative mood amongst the participants. The green urban space was preferred by the participants, as it was calming and quieter than the urban busy section, which was reflected in both the objective EEG measures and subjective scales and interviews. Flowers and the natural aspects of the green space appeared to bring pleasure to the participants and contributed to the calming effect. However, the urban busy space had more negative associations, caused by litter and having to watch more carefully for uneven paving, although we had tried to source a route as free from trip hazards as possible. This would have resulted in participants being more alert and concentrating more fully on their surroundings in the urban environment. As expected, there were more “events” recorded during the walks in urban busy space. This included obstacles such as street furniture, and participants also tended to ask for more instructions to ensure that they were following the correct route. This indicates that busy urban spaces tend to have a higher cognitive load required of participants, potentially leading to more negative mood states.

The social context of the built environment is also important for mood when walking in the urban environment. Participants noted that when they were greeted by others, this generated positive mood states, whilst not being greeted or acknowledged by other people led to them feeling more negative about the environment.

Familiarity was an important aspect in influencing mood during the walk. Those participants that had walked the route before or had visited the testing site before tended to remember positive memories and experiences during the walk, which led to positive mood states. The differences between the Affectiv outputs and the self-report outputs need further consideration, however. While, in some cases, there was alignment in terms of the pattern of responses, there was sometimes a large difference in magnitude (e.g., “frustration” in urban busy to urban green, as shown in Figure 4c). There are a few reasons that this could be the case. Firstly, there is no definitive, published, explanation or definition for what the Affectiv suite terms mean. Secondly, as a consequence of this, these terms were not defined for the participants; they interpreted them in their own way. Therefore, the elicitation interviews were important in understanding if participants considered the terms as positive or negative, especially with terms such as “engagement” and “excitement”, with which there are no obvious positive or negative connotations. An alternative method for future research might be to use established scales (such as the Bond-Lader mood scale [36]) and correlate the Affectiv outputs with these established outputs. Without the definitions of the Affectiv suite terms, the correlations or lack of them are difficult to understand.

A limitation is that the sample size was small and therefore statistical comparisons between walking routes could not been undertaken on the EEG data where a larger sample size would have been analysed using regression analyses. Indeed, the results here appear to be different from the larger total sample analysed using a form of regression and published elsewhere. However, there does appear to be differences between both the Affectiv and self-report measures in each of the study conditions. The profile of each is not a mirror image of the other, i.e., urban busy to urban green graphs do not mirror urban green to urban busy graphs, which may suggest that the sequence of experiencing the two spaces is an important factor. There is an indication that participants exposed to the green space prior to entering the urban busy space during the walk may have benefited from this, as the “frustration” data did not deviate when transitioning from urban green to urban busy. The “frustration” levels shown above are incongruous to what would be expected, but this is perhaps due to the small sample size used. Also important to note is that the majority of participants in our study were used to walking alone in the urban environment, with a couple of participants reporting that they occasionally walk alone. The sample is very engaged in walking, and the results may differ for those that are not used to walking alone.

Aligning the Affectiv suite output with events that had been recorded using the field logger provides some additional context to the environment that the participants experienced during the walk. However, during the interviews, participants could confidently recall how they had felt and describe events that had occurred during the walk. Their responses were considered alongside the field notes that had been made during the walk to observe whether these were accurate reflections. This provided detailed insight into their remembered experience, which revealed some common themes. These included anxiousness upon commencing the walk due to headset visibility, awareness of the experimental set-up, and looking for landmarks during the walk for navigation. In terms of the methodology of the study, there was a one-week break between undertaking the walking session and the interviews, to ensure that participants weren’t overburdened on the testing day. This break may perhaps lead to responses in the interview that came from reflection throughout the preceding week, as opposed to an accurate representation of how they may have felt at the time. However, the reason for using the video as a method of eliciting responses was in order to minimize this [37]. 

This analysis demonstrates the benefit of conducting a video elicitation interview, as researchers can gain insights into the thinking process of participants following their participation in a walk during an EEG experiment. In this context, this process mainly involved thoughts on the physical built environment. However, this in turn this led to discussions about the social aspects of the environment and how participants felt in different urban spaces. The interviews also revealed that the participants were aware they were taking part in a study and needed to follow a particular route. This may explain why “engagement” levels were high in the self-report results for both routes and were also high in the Affectiv suite outputs for the initial section of both walks, irrespective of whether this was an urban green or urban space. This perceived and observed increase in levels of “engagement” suggests that participants may be interacting with their environment in a different way to what they would normally do when not taking part in a research study. The presence of a research assistant during the study appeared to provide reassurance to the participants as the researcher acted as a “landmark” to ensure that they were following the correct route. Particularly for participants starting the walk in the urban busy section, the improvement in mood is considered to be attributed to feeling relieved at seeing the researcher before entering the green space, as this indicated to the participants that they will be entering the green space, as opposed to feeling any emotional connections to the researchers. The researchers did not speak to the participants whilst they were undertaking the walk, with the exception of when participants asked for instructions, and were far away enough not to interfere with the participants’ walking behaviours.

The interview results suggest that the participants eventually become unaware or at least less self-conscious about the headset. Perhaps importantly, participants reported becoming unaware of the headsets irrespective of the route walked. This may seem surprising given that the busy urban area was more populated than the green space, so one might expect participants to be aware of the headset for longer than in the green space. As EEG studies tend to be undertaken in lab settings, this study is one of the first of its kind being taken outdoors. Therefore we wanted to further understand how participants experienced the study to help inform further research protocols for studies outside. Future studies may wish to compare against a controlled environment where there are no or limited numbers of people. This study used a practice session to familiarise participants with the headset and study protocol, but this may not be adequate to prepare participants for the experience of wearing the headset in public. Future studies might then consider undertaking a short public walk as part of the practice session to ameliorate the initial effects in the experimental session. Being and staying mobile in older age has benefits for quality of life [5,38]. However, explicit examinations of mood using neuroimaging methods whilst walking outdoors in changing urban environment types has not been explored in conjunction with qualitative methods. Similarly, there is no research known to the authors that explores the neural effect of walking in urban and natural environments in older participants. Some research suggests that cognitive function is affected by mobility in that there is an increased chance of impaired cognitive function in conjunction with an increased progression of mobility dysfunction in a cohort with a mean age of 68.9 (range 40–100 years) [39]. Thus, the importance of staying mobile, when possible, is of benefit not only for the clear physical benefits but also for psychological benefits. This is supported by research implying that there is a dose-response relationship between negative mood and mobility limitations [40]. 

The methodological addition of using a video as a prompt in conjunction with an EEG headset appeared to be helpful for participants to remember their feelings and experiences. There is evidence to suggest that there are differences in neural processing involved in recall and recollection [41,42,43], so combining a video prompt that elicits a recall of experiences may help induce a more reliable recollection. The interview data therefore adds context to the walk that would not otherwise be gathered from the Affectiv Suite output alone. This method does rely on participants remembering their experiences while the EEG allows for the collection of real-time objective data, undisturbed.

## 5. Conclusions

In this paper we piloted and presented a novel mixed methods study focusing on the changing mood of older people in two different types of urban environment using interpretations of EEG along with subjective scales and retrospective interviews. This approach allows for a deeper understanding of mood variation amongst older people by identifying important aspects of the physical environment, as well as the resulting social environment, which influences how they feel during a walk.

The Affectiv suite data appeared to show interpretable differences between the urban green and urban busy conditions, further supported by the interview analysis, which suggested that participants experienced a beneficial effect of green space. Such evidence shows that urban green space has a role to play in contributing to a supportive city environment for older people through mediating the stress induced by built up settings. However, these data did not always align with the subjective responses that used the same Affectiv Suite terms. Using video elicitation interviews assisted with some interpretation of the Affectiv Suite terms. For older adults, the “engagement” and “excitement” terms were used with both positive and negative associations. However, the “frustration” parameter did not appear to reflect the participants’ experience during the walk as this was interpreted differently by each of the participants. This combination of results suggests that there needs to be caution in the interpretation of Affectiv Suite results for different population groups.

During the interviews, participants were confident in recalling their experience of the walk during the study. The interviews revealed that their remembered experienced aligned well with the field notes collected by researchers on site immediately following the experiment walk. Positive emotional responses were reported during the walk through the green space, whilst there was increased awareness of and sensitivity to urban areas, potentially causing stress amongst our older participants.

The study findings have implications for policies related to age friendly environments and environmental prescribing. The development of age friendly cities has been promoted by the World Health Organization to address the challenges posed by increasing urbanisation and ageing populations and include approaches termed “active ageing” [44]. The physical action of walking has been linked to the concept of “therapeutic mobilities”, enabling health and wellbeing gains [45]. This has been taken up within policy guidance [46] and schemes such as “Walking for Health”, which promotes physical activity for health promotion. Our project findings imply that to maximise the benefits of promoting walking for older people, consideration of routes in terms of patients’ familiarity and preferences needs to be considered. This would include recommendation of routes that preference greenspaces alongside opportunities for social interactions. In addition, policy advice for urban planners [47] needs to further promote accessible green infrastructure within the context of age friendly environments.

Our participant interviews provided insight into interpretations of the Affectiv suite terms for older adults in the context of walking in different urban environments. However, there is still difficulty in establishing reliable interpretations of the Affectiv terms for use in this context, suggesting that further research is required using a larger sample. Given the difficulties in interpretation between the subjective and objective outputs shown here, we recommend future testing of the Affectiv Suite terms against validated subjective emotional scales.

## Figures and Tables

**Table 1:** Summary of weather conditions and participant familiarity with the study site.

Urban Busy to Urban Green			Urban Green to Urban Busy		
Participant	Walked Route before	Weather	Participant	Walked Route before	Weather
1	Y	Grey, misty	1	Y	Cloudy, cold
2	Y	Sunny, warm	2	N	Sunny, warm
3	N	Sunny, cold	3	N	Cloudy, cold
4	Y	Very sunny, warm			
5	N	Very sunny, warm			

Y: Yes; N: No.

**Table 2:** An overview and typology of the quartiles from urban green to urban busy derived from Geographic Information System (GIS).

Section	Typology
UG1	Urban park with a couple of mature trees bordering the path. Most of the trees on both sides are young and protected by cages; there is a single bench and cracked pavement sections
UG2	Urban park with young trees lining the path further apart; there is a single bench, poor pavement sections, and major path intersection
UG3	Urban park with some large trees closer together bordering the path on both sides, benches, cracked pavement sections, major path intersection, and a commemorative plaque
UG4	Urban park with some large trees covering the path, a large pothole in the path, and the park exit
INT	Interchange: road crossing and transitional section between urban green and urban busy
UB1	Wide pavements with residential buildings and a major road crossing leading to narrow pavements with fast food shops and a bus stop
UB2	Narrow pavements with some derelict retail units and good quality surfaces leading to a good yard entrance
UB3	Wide pavements with good quality surfaces and major retail with a bus stop leading to a pinch point and narrow pavements
UB4	Narrow pavements with good quality surfaces and disused retail leading to wide pavements, major retail, a complex road junction, and street trees

**Table 3:** Description of events occurring across the walks during the urban busy to urban green walk.

	Urban Busy				Urban Green			
	Section 1	Section 2	Section 3	Section 4	Section 5	Section 6	Section 7	Section 8
**Field logger results** (events)	Obstacle (1)	Obstacle (1)	Obstacle (1) Pause (1)	Pause (3)		Pause (1)		
				Instruction (1)				
				Road crossing (5)				

**Table 4:** Description of events occurring across the walks during the urban green to urban busy walk.

	Urban Green				Urban Busy			
	Section 1	Section 2	Section 3	Section 4	Section 5	Section 6	Section 7	Section 8
**Field logger results** (events)			Extreme noise (1)	Extreme noise (1)	Pause Road crossing (3)		Instructions (1)	Instructions (1)

## References

[1] World Health Organization (WHO) Mental Health and Older Adults. http://www.who.int/mediacentre/factsheets/fs381/en/.

[2] Scottish Executive (2007). All Our Futures: Planning for a Scotland with an Ageing Population.

[3] Mental Health Foundation (2009). All Things Being Equal: Age Equality in Mental Health Care for Older People in England.

[4] Davidson S., Rossall P. (2014). Age UK Loneliness Evidence Review, Revised July 2014.

[5] Metz D.H. (2000). Mobility of older people and their quality of life. Transp. Policy.

[6] Davey J.A. (2007). Older people and transport: Coping without a car. Ageing Soc..

[7] Van Cauwenberg J., van Holle V., Simons D., Deridder R., Clarys P., Goubert L., Nasar J., Salmon J., de Bourdeaudhuij I., Deforche B. (2012). Environmental factors influencing older adults’ walking for transportation: A study using walk-along interviews. Int. J. Behav. Nutr. Phys. Act..

[8] World Health Organisation (2007). Global Age-Friendly Cities: A Guide.

[9] Velarde M.D., Fry G., Tveit M. (2007). Health effects of viewing landscapes—Landscape types in environmental psychology. Urban For. Urban Green..

[10] Velarde M.D., Tveit M.S., Hagerhall C.M. (2010). The Link between Landscape Preferences and Perceived Restorativeness—Current Research Trends and Suggestions for Future Studies. Environmental Psychology: New Developments.

[11] Bratman G.N., Hamilton J.P., Hahn K.S., Daily G.C., Gross J.J. (2015). Nature experience reduces rumination and subgenual prefrontal cortex activation. Proc. Natl. Acad. Sci. USA.

[12] Gidlow C.J., Jones M.V., Hurst G., Masterson D., Clark-Carter D., Tarvainen M.P., Smith G., Nieuwenhuijsen M. (2016). Where to put your best foot forward: Psycho-physiological responses to walking in natural and urban environments. J. Environ. Psychol..

[13] Berman M.G., Jonides J., Kaplan S. (2008). The cognitive benefits of interacting with nature. Psychol. Sci..

[14] Berman M.G., Kross E., Krpan K.M., Askren M.K., Burson A., Deldin P.J., Kaplan S., Sherdell L., Gotlib I.H., Jonides J. (2012). Interacting with nature improves cognition and affect for individuals with depression. J. Affect. Disord..

[15] Takano T., Nakamura K., Watanabe M. (2002). Urban residential environments and senior citizens’ longevity in megacity areas: The importance of walkable green spaces. J. Epidemiol. Community Health.

[16] Astell-Burt T., Feng X., Kolt G.S. (2013). Mental health benefits of neighbourhood green space are stronger among physically active adults in middle-to-older age: Evidence from 260,061 Australians. Prev. Med..

[17] Kaplan S. (1995). The restorative benefits of nature: Toward an integrative framework. J. Environ. Psychol..

[18] Mulckhuyse M., Theeuwes J. (2010). Unconscious attentional orienting to exogenous cues: A review of the literature. Acta Psychol..

[19] Kaplan R., Kaplan S. (1989). The Experience of Nature: A Psychological Perspective.

[20] Kaplan S. (2001). Meditation, restoration, and the management of mental fatigue. Environ. Behav..

[21] Chang C.Y., Hammitt W.E., Chen P., Machnik L., Su W. (2008). Psychophysiological responses and restorative values of natural environments in Taiwan. Landsc. Urban Plan..

[22] Kim G.W., Jeong G., Kim T., Baek H., Oh S., Kang H., Lee S., Kim Y.S., Song J. (2010). Functional neuroanatomy associated with natural and urban scenic views in the human brain: 3.0T functional MR imaging. Korean J. Radiol..

[23] Kim T.H., Jeong G.W., Baek H.S., Kim G.W., Sundaram T., Kang H.K., Lee S.W., Kim H.J., Song J.K. (2010). Human brain activation in response to visual stimulation with rural and urban scenery pictures: A functional magnetic resonance imaging study. Sci. Total Environ..

[24] Martínez-Soto J., Gonzales-Santos L., Pasaye E., Barrios F.A. (2013). Exploration of neural correlates of restorative environment exposure through functional magnetic resonance. Intell. Build. Int..

[25] Ulrich R.S. (1981). Natural Versus Urban Scenes: Some Psychophysiological Effects. Environ. Behav..

[26] Aspinall P., Mavros P., Coyne R., Roe J. (2013). The urban brain: Analysing outdoor physical activity with mobile EEG. Br. J. Sports Med..

[27] Jewitt C. (2012). An Introduction to Using Video for Research. NCRM Working Paper.

[28] Sperry R.W. (1968). Hemisphere deconnection and unity in conscious awareness. Am. Psychol..

[29] Cacot P., Tesolin B., Sebban C. (1995). Diurnal variations of EEG power in healthy adults. Electroencephalogr. Clin. Neurophysiol..

[30] Cummings L., Dane A., Rhodes J., Lynch P., Hughes A.M. (2000). Diurnal variation in the quantitative EEG in healthy adult volunteers. Br. J. Clin. Pharmacol..

[31] Mavros P., Skrompelou K. Fieldworker. https://github.com/pmavros/Fieldworker.

[32] City of Edinburgh Council, P.A.B.S. Planning Information, Services for Communities (2013). Population Distribution and Density in Edinburgh: Recent Trends and Comparisons with Other Cities Across Scotland and the UK.

[33] CrashMap About Us. http://www.crashmap.co.uk/Home/AboutUs.

[34] Braun V., Clarke V. (2006). Using thematic analysis in psychology. Qual. Res. Psychol..

[35] Bradford J.C., Lukos J.R., Ferris D.P. (2016). Electrocortical activity distinguishes between uphill and level walking in humans. J. Neurophysiol..

[36] Bond A., Lader M. (1974). The use of analogue scales in rating subjective feelings. Br. J. Med. Psychol..

[37] Henry S.G., Fetters M.D. (2012). Video elicitation interviews: A qualitative research method for investigating physician-patient interactions. Ann. Fam. Med..

[38] Banister D., Bowling A. (2004). Quality of life for the elderly: The transport dimension. Transp. Policy.

[39] Tolea M.I., Galvin J.E. (2016). The relationship between mobility dysfunction staging and global cognitive performance. Alzheimer Dis. Assoc. Disord..

[40] Hirvensalo M., Sakari-Rantala R., Kallinen M., Leinonen R., Lintunen T., Rantanen T. (2007). Underlying factors in the association between depressed mood and mobility limitation in older people. Gerontology.

[41] Gillund G., Shiffrin R.M. (1984). A retrieval model for both recognition and recall. Psychol. Rev..

[42] Snodgrass J.G., Vanderwart M. (1980). A standardized set of 260 pictures: Norms for name agreement, image agreement, familiarity, and visual complexity. J. Exp. Psychol. Hum. Learn. Mem..

[43] Yonelinas A.P. (2002). The nature of recollection and familiarity: A review of 30 years of research. J. Mem. Lang..

[44] Steels S. (2015). Key characteristics of age-friendly cities and communities: A review. Cities.

[45] Gatrell A.C. (2013). Therapeutic mobilities: Walking and“steps” to wellbeing and health. Health Place.

[46] National Institute for Health and Care Excellence (2012). Physical Activity: Walking and Cycling.

[47] Natural England (2009). Green Infrastructure Guidance.

